# The expression dynamics of transforming growth factor-β/Smad signaling in the liver fibrosis experimentally caused by *Clonorchis sinensis*

**DOI:** 10.1186/s13071-015-0675-y

**Published:** 2015-02-04

**Authors:** Chao Yan, Lin Wang, Bo Li, Bei-Bei Zhang, Bo Zhang, Yan-Hong Wang, Xiang-Yang Li, Jia-Xu Chen, Ren-Xian Tang, Kui-Yang Zheng

**Affiliations:** Department of Pathogenic Biology and Immunology, Laboratory of Infection and Immunity, Xuzhou Medical College, Xuzhou, Jiangsu Province 221004 PR China; Laboratory of Parasite and Vector Biology, National Institute of Parasitic Diseases, Chinese Center for Disease Control and Prevention, Shanghai, 200025 PR China

**Keywords:** *Clonorchis sinensis*, Liver fibrosis, Transforming growth factor-β, Smads

## Abstract

**Background:**

Liver fibrosis is a hallmark of clonorchiasis suffered by millions people in Eastern Asian countries. Recent studies showed that the activation of TGF-β/Smad signaling pathway can potently regulate the hepatic fibrogenesis including *Schistosoma* spp. and *Echinococcus multilocularis*-caused liver fibrosis. However, little is known to date about the expression of transforming growth factor-β (TGF-β) and other molecules in TGF-β/Smad signaling pathway which may play an important role in hepatic fibrosis caused by *C. sinensis.*

**Methods:**

A total of 24 mice were individually infected orally with 45 metacercariae, both experimental mice and mocked-infected control mice were anesthetized at 4 week post-infection (wk p.i.), 8 wk p.i. and 16 wk p.i., respectively. For each time-point, the liver and serum from each animal were collected to analyze histological findings and various fibrotic parameters including TGF-β_1_, TGF-β receptors and down-stream Smads activation, as well as fibrosis markers expression.

**Results:**

The results showed that collagen deposition indicated by hydroxyproline content and Masson’s trichrome staining was increased gradually with the development of infection. The expression of collagen type α1 (Col1a) mRNA transcripts was steadily increased during the whole infection. The mRNA levels of Smad2, Smad3 as well as the protein of Smad3 in the liver of *C. sinensis*-infected mice were increased after 4 wk p.i. (*P* < 0.05, compared with normal control) whereas the TGF-β_1_, TGF-β type I receptor (TGFβRI) and TGF-β type II receptor (TGFβRII) mRNA expression in *C. sinensis*-infected mice were higher than those of normal control mice after 8 wk p.i. (*P* < 0.05). However, the gene expression of Smad4 and Smad7 were peaked at 4 wk p.i. (*P* < 0.05), and thereafter dropped to the basal level at 8 wk p.i., and 16 wk p.i., respectively. The concentrations of TGF-β_1_ in serum in the *C. sinensis*-infected mice at 8 wk p.i. and 16 wk p.i (*P* < 0.05) were significantly higher than those in the control mice.

**Conclusions:**

The results of the present study indicated for the first time that the activation of TGF-β/Smad signaling pathway might contribute to the synthesis of collagen type I which leads to liver fibrosis caused by *C. sinensis*.

## Background

*Clonorchis sinensis* is a food-borne zoonotic parasite, which is epidemic in some Eastern Asian countries including China, Korea, Japan, and Vietnam. In humans, it is assumed that approximately 15–20 million people were suffering from clonorchiasis whereas the number of infected people was 12.5 million in China according to a report based on a nationwide survey [1,2]. Human become infected by ingestion of freshwater fish containing *C. sinensis* metacercariae. The metacercariae develop into *C. sinensis* juveniles in the duodenum by the stimulation of trypsin, and then rapidly move to the intrahepatic bile duct where the juvenile worms become mature and survive for decades [3,4]. *C. sinensis* infection can induce significant cholangitis, adenomatous hyperplasia mechanical obstruction of the hepatobiliary duct and cholelithiasis [5]. Furthermore, *C. sinensis* is considered as a group I carcinogen-metazoan parasite to potentially induce cholangiocarcinoma in humans [6]. Chronic infection with *C. sinensis* can also potently lead to liver fibrosis which is marked with excessive accumulation of extracellular matrix components (ECM) due to an imbalance between its synthesis and degradation [2,7,8]. Moreover, some components of worms and its excretory/secretory products (ESP) which can probably participate in the development of hepatic fibrosis have been widely investigated in the lab of Professor Yu [9-13]. However, molecular mechanism underlying fibrotic responses of hosts to these virulence factors is not fully elucidated.

Transforming growth factor-β (TGF-β) as one of major pro-fibrotic cytokines plays a crucial role in orchestrating fibrogenesis and it is demonstrated that active TGF-β_1_ motivates its downstream signaling pathway, leading to phosphorylation of Smad2 and Smad3 (also called R-Smad) which is meditated by TGF-β type I (TGFβRI) and type II receptors (TGFβRII), phosphorylated Smad2 and Smad3 rapidly combine with a common mediator called Smad4 and subsequently migrates to the nucleus, resulting in massive fibrotic genes expression (such as collagen type I) [14-17]. TGF-β/Smad signaling pathway has been proved as a canonical pathway that can potently regulate the hepatic fibrogenesis [18,19], and a few studies have addressed about the activation of TGF-β/Smad signaling in fibrogenesis caused by parasitic infection, such as *Schistosoma* spp. and *Echinococcus multilocularis*, which suggested that TGF-β/Smad signaling play important roles in the development of liver fibrosis [20-22]. However, to our best knowledge, little is known of the expression and potential roles of TGF-β/Smad signaling pathway which may be involved in process of hepatic fibrosis caused by *C. sinensis*. In the light of this background, the objectives of the present study were to investigate the expression dynamics of TGF-β/Smad pathway and analyze their possible roles in the development of hepatic fibrosis in BALB/c mice infected by *C. sinensis.*

## Methods

### Parasites

*Pseudorasbora parva*, the second intermediate hosts which were naturally infected with *C. sinensis*, were collected in Guangxi Autonomous Region, People’s Republic of China. And the fish were transported to our laboratory by air. Metacercariae of *C. sinensis* were collected by digesting fish with a pepsin-HCl (0.6%) artificial gastricjuice. The collected metacercariae were preserved in cold Alsever’s solution with antibiotics until use.

### Animals

Female BABL/c mice (6 ~ 8 weeks old, 22 ± 2 g) were purchased from Shanghai Laboratory Animal Co., Ltd (SLAC, Shanghai, China). The mice were housed in an air-conditioned room at 24°C with a 12 h dark/light cycle and permitted free access to standard laboratory food and water. All animal experiments were approved by the Animal Care and Use Committee of Xuzhou Medical Collage. The mice were individually infected orally with 45 metacercariae. Mock-infected control mice were similarly administered with 50 μl of sterile normal solution. Both experimental mice (n = 24) and control mice (n = 15) were divided into 3 groups and anesthetized at 4 week post-infection (wk p.i.), 8 wk p.i. and 16 wk p.i., respectively. For each time-point, the liver and serum from each animal were harvested to analyze histological findings and various fibrotic parameters.

### Histological examination and evaluation of hepatic fibrosis in mice caused by *C. sinensis*

For histological evaluation, all liver samples were fixed in formalin, embedded in paraffin. 4 μm thick sections were prepared and then stained with hematoxylin and eosin (H&E) and Masson’s trichrome (MT). These specimens were observed and photographed under an inverted microscope. Collagen depositions from 5–8 images of each specimen were quantified using Image-Pro Plus software (Media Cybernetics, Rockville, MD, USA).

### Determination of hydroxyproline content

Collagen was also determined by evaluating the concentration of the hydroxyproline (Hyp), an amino acid characteristic of collagen. The lysates were used to measure hydroxyproline contents using commercially available kits according to the manufacturer’s instructions (Jiancheng Institute of Biotechnology, Nanjing, China). In this kit, hydroxyproline concentration was determined by the reaction of oxidized hydroxyproline with 4-(Dimethylamino) benzaldehyde (DMAB), which was measured spectrophotometrically at 560 nm.

### Quantitative real-time PCR analysis

Total RNA was extracted from liver tissues using TRIzol reagent (TIANGEN Biotech, Beijing, China) as described by the manufacturer. RNA was reverse-transcribed using the Reverse Transcription Kit (TIANGEN Biotech, Beijing, China). To investigate the expression of TGF-β/Smad pathway in the liver, relative quantitative RT-PCR (qPCR) was performed using the LightCycler FastStart DNA Master SYBR Green I kit (Roche Applied Science, Mannheim, Germany) according to the manufacturers’ protocol with primer sequences shown in Table 1. The optimal light cycler conditions were: initial denaturation at 95°C for 5 min, followed by 40 cycles with denaturation at 95°C for 30 s, annealing at 60°C for 30 s and elongation at 72°C for 30 s (Table 1). Quantification of target gene expression was evaluated in the terms of the comparative cycling threshold (C_t_) normalized by β-actin with the 2^-△△Ct^ method.

**Table 1:** **Primers used in the present study**

**Gene**	**Genbank accession**	**Primer sequences**	**Annealing temperature**	**Expected size (bp)**	**Ref.**
α-SMA	NM_007392.3	F:5′-AAGAGCATCCGACACTGCTGAC-3′	60.0°C	300	Present study
		R:5′-AATAGCCACGCTCAGTCAGG-3′			
ColIa	NM_007742.3	F: 5′-CAGGGTATTGCTGGACAACGTG-3′	60.0°C	107	Present study
		R: 5′-GGACCTTGTTTGCCAGGTTCA-3′			
Col-III	NM_009930.2	F: 5′-TGGCACAGCAGTCCAACGTA-3′	60.0°C	122	Present study
		R: 5′-AAGGACAGATCCTGAGTCACAGACA-3′			
TGF-β_1_	NM_011577	F: 5′-GTGTGGAGCAACATGTGGAACTCTA-3′	60.0°C	143	20
		R: 5′-TTGGTTCAGCCACTGCCGTA-3′			
TGFβRI	NM_009370.2	F: 5′-TGCAATCAGGACCACTGCAATAA-3′	60.0°C	133	20
		R: 5′-GTGCAATGCAGACGAAGCAGA-3′			
TGFβRII	NM_009371.2	F: 5′-AAATTCCCAGCTTCTGGCTCAAC-3′	60.0°C	100	20
		R: 5′-TGTGCTGTGAGACGGGCTTC-3′			
Smad2	NM_010754	F: 5′-TGCATTCTGGTGTTCAATCG-3′	60.0°C	198	20
		R: 5′-CGAGTTTGATGGGTCTGTGA-3′			
Smad3	NM_016769	F: 5′-GTCAACAAGTGGTGGCGTGTG-3′	60.0°C	150	20
		R: 5′-GCAGCAAAGGCTTCTGGGATAA-3′			
Smad4	NM_008540	F: 5′-TGACGCCCTAACCATTTCCAG-3′	60.0°C	136	20
		R: 5′-CTGCTAAGAGCAAGGCAGCAAA-3′			
Smad7	NM_001042660.1	F: 5′-AGAGGCTGTGTTGCTGTGAATC-3′	60.0°C	126	20
		R: 5′-CCATTGGGTATCTGGAGTAAGGA-3′			
β-actin	NM_007393.3	F: 5′-CGTGGGCCGCCCTAGGCACCA-3′	60.0°C	243	Present study
		R: 5′-TTGGCCTTAGGGTTCAGGGGGG-3′			

### Western blot

Total protein was extracted from liver tissues and analyzed with bicinchoninic acid protein concentration assay kit (Beyotime Biotech, Beijing, China). Sample protein was separated by electrophoresis in 12% SDS-PAGE with a Bio-Rad electrophoresis system (Hercules, CA, USA). The primary antibodies (rabbit anti-Smad3 antibody, UCallM biotech Co., Ltd, Wuxi, China, 1:1000 dilutions) were incubated at 4°Covernight. The corresponding horseradish-peroxidase-conjugated secondary antibodies (anti-rabbit IgG, 1:5000 dilutions) were incubated for 1 h at room temperature. The membrane containing antibody-protein complexes were visualized with an enhanced chemiluminescence detection system on radiograph film. The brands were scanned and analyzed by the software Quantity ONE (Bio-rad, Hercules, CA, USA). The expression of protein in each sample was normalized by α-Tublin(Santa Cruz Biotechnology, CA, USA).

### Enzyme linked immunosorbent assay (ELISA)

Serum from each mouse was immediately used to evaluate the concentration of TGF-β_1_ by a specific ELISA kit (eBiosciences, CA, USA). In brief, samples were firstly activated by 1 mol/L HCl, and then samples as well as serial dilutions of standards were added to 96-well plates pre-coated with anti-TGF-β_1_ and pre-blocked with PBS containing 10% fetal bovine serum (FBS), after samples were washed, horseradish peroxidase (HRP)-conjugated streptavidin A in PBS containing 10% FBS was added for 30 min at room temperature. After final washes, the HRP substrate TMB (3,3,5,5-tetramethylbenzidine) was added, and the optical density of the color reaction was measured at 450 nm. Concentrations of cytokine in the sera were calculated using standard curves as references.

### Data analysis

All values were expressed as mean ± SEM. Comparisons between control and each experimental group were made by one-way analysis of variance (ANOVA) and Student’s unpaired t-test using the SPSS 13.0 statistical package. Differences were considered statistically significant at *P* < 0.05.

## Results

### Histological findings and evaluation of hepatic fibrosis in mice caused by *C. sinensis*

**Figure 1:**
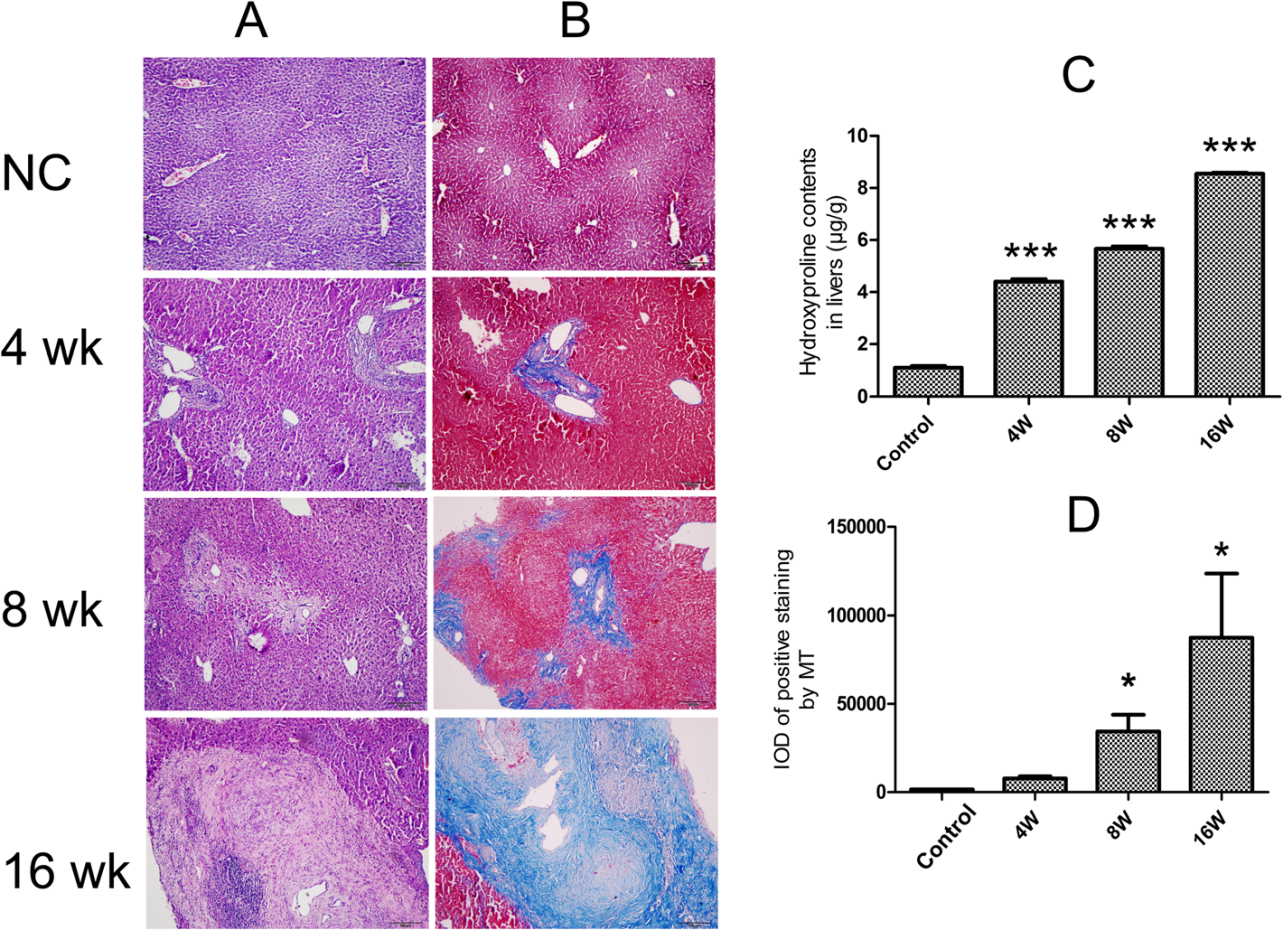
**Evaluation of hepatic fibrosis in mice caused by** ***Clonorchis sinensis*** **at 4 week post-infection (wk p.i.), 8 wk p.i. and 16 wk p.i. (A)** Histological examination of liver tissues from *C. sinensis-*infected mice and normal control mice at different time-points as indicated. **(B)** Collagen depositions were specifically stained by Masson’s trichrome at different time-points as indicated. **(C)** Hydroxyproline (Hyp) concentration was measured in liver homogenate (0.1 g) in fibrotic or normal livers of BALB/c mice at indicated time-points. **(D)** Collagen depositions from each specimen were semi-quantified using Image-Pro Plus software. Data were presented as mean ± SEM from 8 *C. sinensis*-infected mice and 5 normal control (NC) mice at each time-point, * = *P* < 0.05, ** = *P* < 0.01, *** = *P* < 0.001 versus control mice.

In the normal control group, the hepatocyte arranged tightly and hepatic lobules were observed completely. In contrast, in the *C. sinensis*-infected group, fibrotic cords were observed in the periportal areas of *C. sinensis*- infected mice at 4 wk p.i. and 8 wk p.i., and as the infection developed, collagen fibers were extended from portal areas to liver lobule of mice at 16 wk p.i., the arrangement of hepatocyte was disordered and pseudolobules were observed in some serious cases at this time point (Figure 1A and Figure 1B). The quantities of collagen depositions were increased gradually with the development of infection, and statistical difference and significant difference were observed at 8 wk p.i. (*P* < 0.05), and 16 wk p.i. (*P* < 0.05), respectively, compared with normal control mice (Figure 1D).

The hydroxyproline which is a major component of collagen was also evaluated as an indicator of collagen content. Compared with normal control mice, the levels of hydroxyproline were significantly augmented at 4 wk p.i. (*P* < 0.001), and thereafter dramatically increased at 8 wk p.i. (*P* < 0.001) and 16 wk p.i. (*P* < 0.001, Figure 1C).

### The expression of the pro-fibrotic molecular markers in livers of mice during *C. sinensis* infection

**Figure 2:**
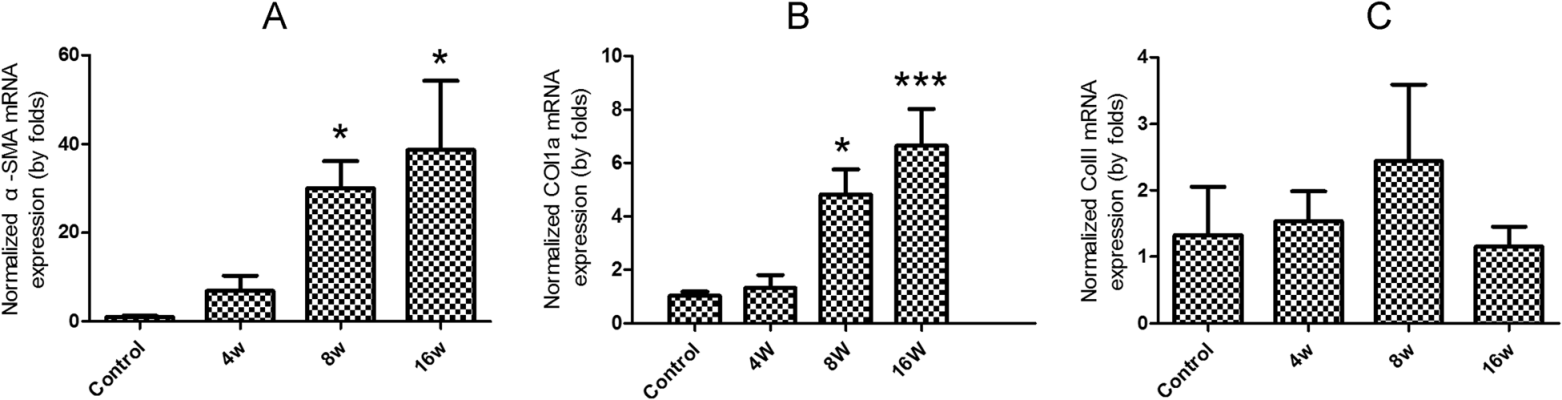
**The mRNA expression of the pro-fibrotic molecular markers in livers of mice during** ***Clonorchis sinensis*** **infection.** Mice were orally infected with 45 metacercariae of *C. sinensis*, livers were collected from infected and non-infected mice at indicated time-points and mRNA levels were determined by qPCR (normalized to beta-actin transcript levels). **(A)** α-SMA; **(B)** Col1a; **(C)** Col III. Data were presented as mean ± SEM from 8 *C. sinensis*-infected mice and 5 normal control mice at each time-point, * = *P* < 0.05, ** = *P* < 0.01, *** = *P* < 0.001 *versus* control mice.

qPCR results showed that the mRNA level of alpha-smooth muscle actin (α-SMA) was dramatically increased from 4 wk p.i. to 16 wk p.i., and there were statistical differences for α-SMA mRNA expression between 8 wk p.i. or 16 wk p.i. and control animals (*P* < 0.05, Figure 2A). The results also showed that mRNA levels of collagen α1(Col1a) expression were steadily increased from 4 wk p.i. to 16 wk p.i., and significant differences were found at 8 wk p.i. (*P* < 0.05) and 16 wk p.i. (*P* < 0.05), compared with normal control animals (Figure 2B). However, collagen type III (Col III) expression was not changed at 4 wk p.i., 8 wk p.i. or 16 wk p.i., and there were no statistical differences between *C. sinensis*-infected animals and normal control ones (Figure 2C, *P* > 0.05).

### The mRNA expression of TGF-β/Smad in the liver of BALB/c during *C. sinensis*-infection

**Figure 3:**
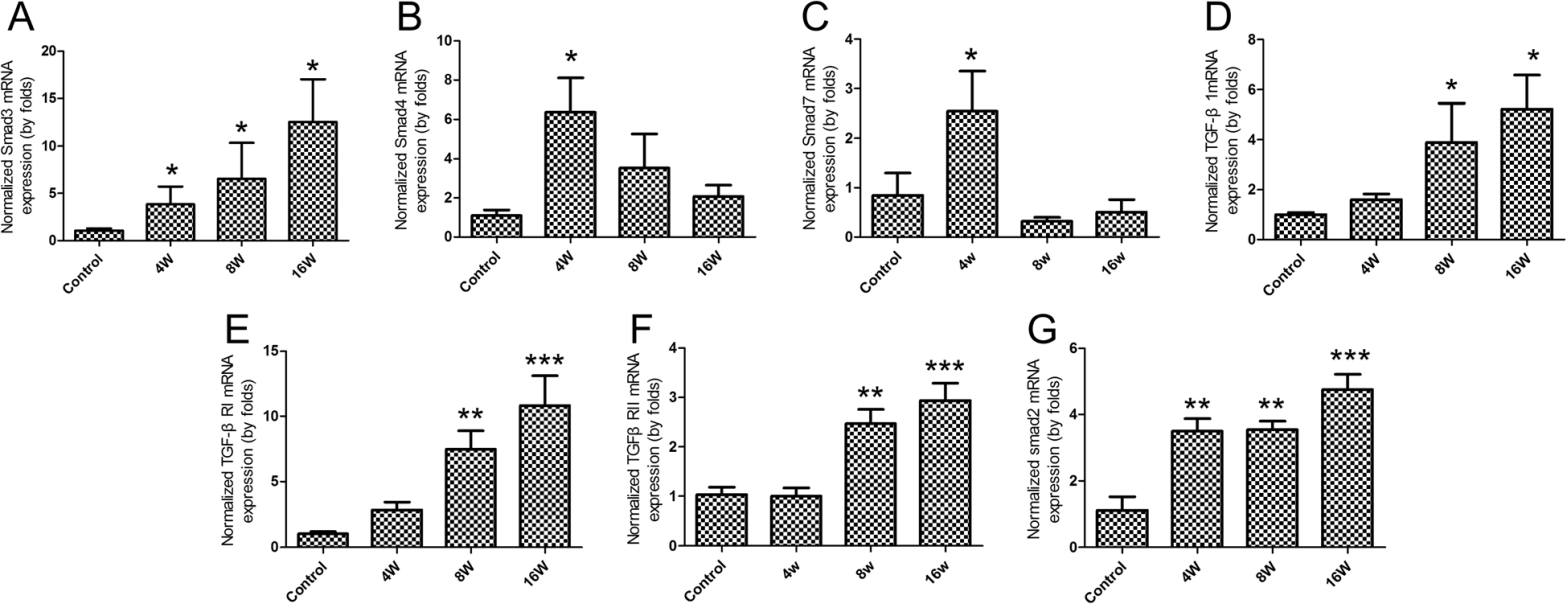
**Gene expression of TGF-β/Smad signaling in the liver fibrosis caused by** ***C. sinensis*** **.** Mice were orally infected with 45 metacercariae of *C. sinensis*, livers were collected from infected and non-infected mice at indicated time-points and mRNA levels were determined by qPCR (normalized to beta-actin transcript levels). **(A)** Smad3; **(B)** Smad4; **(C)** Smad7; **(D)** TGF-β_1_; **(E)** TGFβRI; **(F)** TGFβRII; **(G)** Smad2. Data were presented as mean ± SEM from 8 *C. sinensis*-infected mice and 5 normal control mice at each time-point, * = *P* < 0.05, ** = *P* < 0.01, *** = *P* < 0.001 *versus* control mice.

As shown in Figure 3, mRNA expression of TGF-β_1_, TGFβRI, TGFβRII, Smad2 and Smad3 were upregulated in the liver of *C. sinensis*-infected mice, compared with normal control mice. And significant differences were observed from 8 wk p.i. to 16 wk p.i. for TGF-β_1_, TGFβRI, TGFβRII (*P* < 0.05), whereas the mRNA expression levels of Smad2 and Smad3 showed statistical differences from 4 wk p.i. to 16 wk p.i. compared with normal control mice (*P* < 0.05). However, Smad4 and Smad7 mRNA peaked at 4 wk p.i., thereafter decreased at 8 wk p.i. and 16 wk p.i. and there was a significant difference between *C. sinensis*-infected mice and control group at 4 wk p.i. (*P* < 0.05).

### The protein expression of Smad3 in livers of *C. sinensis*-infected mice and dynamic changes of serous TGF-β_1_ in mice infected by *C. sinensis*

**Figure 4:**
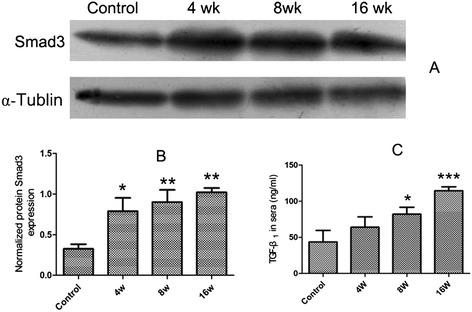
**The protein expression of Smad3 in livers and dynamic changes of serous TGF-β**_**1**_ **in sera from** ***C. sinensis*** **-infected and non-infected mice** ***.*** Mice were orally infected with 45 metacercariae of *C. sinensis*, livers and sera were collected from infected and non-infected mice at indicated time-points and the protein expression levels of Smad3 **(A&B)** in the livers and TGF-β_1_ **(C)** in the sera from mice were determined by western-blot and ELISA, respectively. Data were presented as mean ± SEM from 8 *C. sinensis*-infected mice and 5 normal control mice at each time-point, * = *P* < 0.05, ** = *P* < 0.01, *** = *P* < 0.001 *versus* control mice.

The Smad3 protein in the liver of *C. sinensis*-infected mice was increased dramatically as the infection developed, which was consistent with the mRNA expression of Smad3. Statistical differences were found in expression of Smad3 in all *C. sinensis*-infected groups, compared with normal control group (Figure 4A and Figure 4B, *P* < 0.05).

As shown in Figure 4C, during the whole experimental infection, the concentration of TGF-β_1_ in the serum at 8 wk p.i. and 16 wk p.i. was significantly higher than that of in the control mice (*P* < 0.05), and the level of TGF-β_1_ were increased from 4 wk p.i. to 16 wk p.i during the development of infection.

## Discussion

Previous studies showed that liver fibrosis was orchestrated by a complex network of signaling pathways involved in regulation the deposition of extracellular matrix, and of these signaling pathways, TGF-β/Smad signaling pathway is considered as the most prominent mediator in accelerating liver fibrosis [18,23]. Beside TGF-β, IL-13 and Il-17 were recently demonstrated as another critical pro-fibrotic cytokines in liver fibrosis, for example, IL-13 can potently induce the synthesis of collagen I and other fibrotic markers directly in *Schistosoma* spp. caused liver fibrosis whereas IL-17A can promote the activation of HSC and drive the mRNA expression of the IL-6, α-SMA, collagen, as well as TGF-β_1_ in carbon tetrachloride–induced liver fibrosis [24-27]. However, little is known of the molecular mechanism underlying *C. sinensis* caused liver fibrosis. In the present study, we used BALB/c mice to explore the possible mechanism underlying the liver fibrosis caused by *C. sinensis*. Similar with other study, we showed that BALB/c mice can develop a moderate periductal fibrosis at 4 wk p.i. and massive deposition of extracellular matrix after 8 wk p.i. demonstrated by HE and MT staining, suggesting that the mouse model for *C. sinensis* induced-liver fibrosis in the present study was established successfully [7,8].

Biological functions of TGF-β and its roles in regulating ECM deposition have been intensively reviewed, and proteins of the Smads family members are important mediators that transduce signals induced by TGF-β to specific target genes in the nucleus, leading to the expression of pro-fibrotic genes [28,29]. There were also some studies suggesting that TGF-β_1_ and its downstream Smads played a central role in parasite-induced liver fibrosis [20,30,31], for example, in *Schistosoma mansoni*-infected mouse, the increased expression of TGF-β_1_ and its receptors led to extensive accumulation of extracellular matrix proteins and treatment with anti-fibrotic drugs like praziquantel can reduce the concentration of TGF-β signicantly and led to an reversible liver fibrosis in *S. mansoni*-infected mice [32,33].

As expected, hepatic mRNA transcriptions of fibrotic markers such as collagen type I (Col1a), TGF-β_1_, α-SMA and hydroxyproline contents were increased with the degree of *C. sinensis*-caused hepatic fibrosis, which was indicated by MT staining. To further investigate whether TGF-β/Smad signaling was activated during *C. sinensis*-caused liver fibrosis or not, the expression of genes and proteins within TGF-β/Smad signaling pathway was examined. In the present study, the mRNA expression of TGF-β_1_, Smad2/3, TGFβRI and TGFβRII was significantly stronger in the livers of *C. sinensis* infected-mice than that in control ones, and these changes were positively correlated with the degrees of hepatic fibrosis (data is not shown), suggesting that TGFβ/Smad signaling pathway may be involved in the development of liver fibrosis due to *C. sinensis* infection. However, our study showed Smad4 was found increased at 4 wk p.i., and its expression decreased with the development of hepatic fibrosis, which suggested that the effects of Smad4 might occur at early and middle stage of hepatic fibrosis [34]. Our results also showed that Smad7 expression heightened only at the 4 wk p.i. and the expression of Smad2 was significantly higher in the livers of *C. sinensis*-infection mice, indicating that Smad7 played a negative role in fine-tuning of TGF-β signals since the increased Smad2 may suppress the expression of Smad7 conversely [35,36].

## Conclusions

In conclusion, our results of the present study suggested, for the first time, that expression dynamics of TGF-β/Smad signaling pathway may be involved in the development of hepatic fibrosis caused by *C. sinensis*. The further studies should be warranted to elucidate detailed roles of TGF-β/Smad signaling pathway in *C. sinensis* caused liver fibrosis, which may provide basic information for control clonorchiasis.
